# Endocuticle is involved in caste differentiation of the lower termite

**DOI:** 10.1093/cz/zoab005

**Published:** 2021-01-30

**Authors:** Chenxu Ye, Zhuanzhuan Song, Taoyu Wu, Wenxiu Zhang, Noor us Saba, Lianxi Xing, Xiaohong Su

**Affiliations:** 1 Shaanxi Key Laboratory for Animal Conservation, Northwest University, Xi’an 710069, China; 2 College of Life Sciences, Northwest University, Xi’an 710069, China

**Keywords:** adaptive evolution, caste differentiation, endocuticle, gene expression, transcriptomes

## Abstract

Caste differentiation in termites is one of the most conspicuous examples of facultative polyphenism in animals. It is clear that specific cuticular formation occurs in hard exocuticles during caste differentiation. However, the developmental pattern of the soft endocuticle in the differentiation pathways of castes is unknown. To reveal whether the endocuticle is involved in caste differentiation, we compared the exocuticle and endocuticle thickness of individuals in 2 pathways (nymph line and worker line) of caste differentiation in the termite *Reticulitermes aculabialis*. The endocuticle protein genes were identified by transcriptome analysis and the expression patterns of these genes were confirmed in caste differentiation. We found that the endocuticle structure showed dynamic changes in 2 pathways, and the first difference in endocuticle structure occurred after larvae differentiation bifurcated into workers and nymphs. The thinning of the endocuticle was a significant event from nymphs developing into alates with the thickest exocuticle and thinnest endocuticle. The thickest endocuticle layers were found in the heads of the workers and the ultrastructure of the endocuticle in the heads was more complex than that in the thorax–abdomens. Six endocuticle protein genes were identified and annotated as endocuticle structural glycoproteins SgAbd-2, SgAbd-9, and Abd-5. The expression levels of endocuticle protein genes changed dramatically during caste development and the expression levels in neotenic reproductives (secondary reproductives) were significantly higher than those in alates (primary reproductives). These results reveal the roles of endocuticles in caste differentiation and adaptation to the environment.

Caste differentiation in termites is one of the most conspicuous examples of facultative polyphenism in animals, in which individuals show various phenotypes despite the same genetic background. Colony efficiency is based on the division of labour leading to the allocation of specific tasks and behavioral specializations in different castes. Termites normally possess 3 castes with specific phenotypes (workers, soldiers, and reproductives). The genus *Reticulitermes* within Rhinotermitidae, as lower termites, is characterized by unique flexibility in development (Korb and Hartfelder 2008; Korb et al. 2009; Korb 2015). In *Reticulitermes*, caste differentiation bifurcates during development into 2 pathways: the sexual imaginal or nymphal line, which generally leads to alate adults (primary reproductives), and the apterous line (worker line), which generally leads to workers (Figure 1). The workers are characterized by great flexibility in that a worker can develop in one of 3 ways: remain as a worker, become a soldier, or become a neotenic reproductive (secondary reproductive) developing in the absence of the primary reproductive, helping to provide for continued or additional growth of the colony (Thorne 1999; Su et al. 2017). The morphological and anatomical adaptations are caste-specific in reproductives (to allow dispersal, pair bonding, and fecundity), workers (foraging and feeding, tending and feeding of immatures, nest construction), and soldiers (only defense) (Figure 2). Termite castes have distinctive morphological phenotypes compared with other social insects. However, understanding of the developmental mechanisms of caste-specific phenotypes is still limited (Watanabe et al. 2014; Masuoka and Maekawa 2016). We believe that the different functions of castes are related to exomorphic aspects. However, we know little about the relationship between internal structure and caste differentiation and how the internal structures of individuals change during caste development.

**Figure 1.**
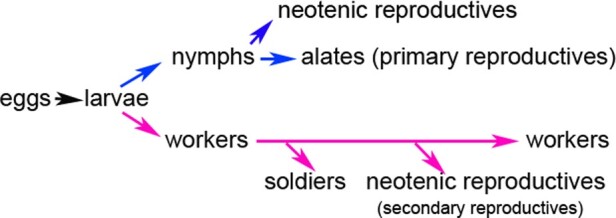
Developmental pathway in *R. aculabialis*. Caste differentiation bifurcates into 2 pathways: the sexual nymph line, which generally leads to alate adults (primary reproductives), and the worker line, which generally leads to workers. The workers are characterized by great flexibility in that a worker can develop in 1 of 3 ways: remain as a worker, become a soldier, or become a neotenic reproductive (secondary reproductives).

**Figure 2.**
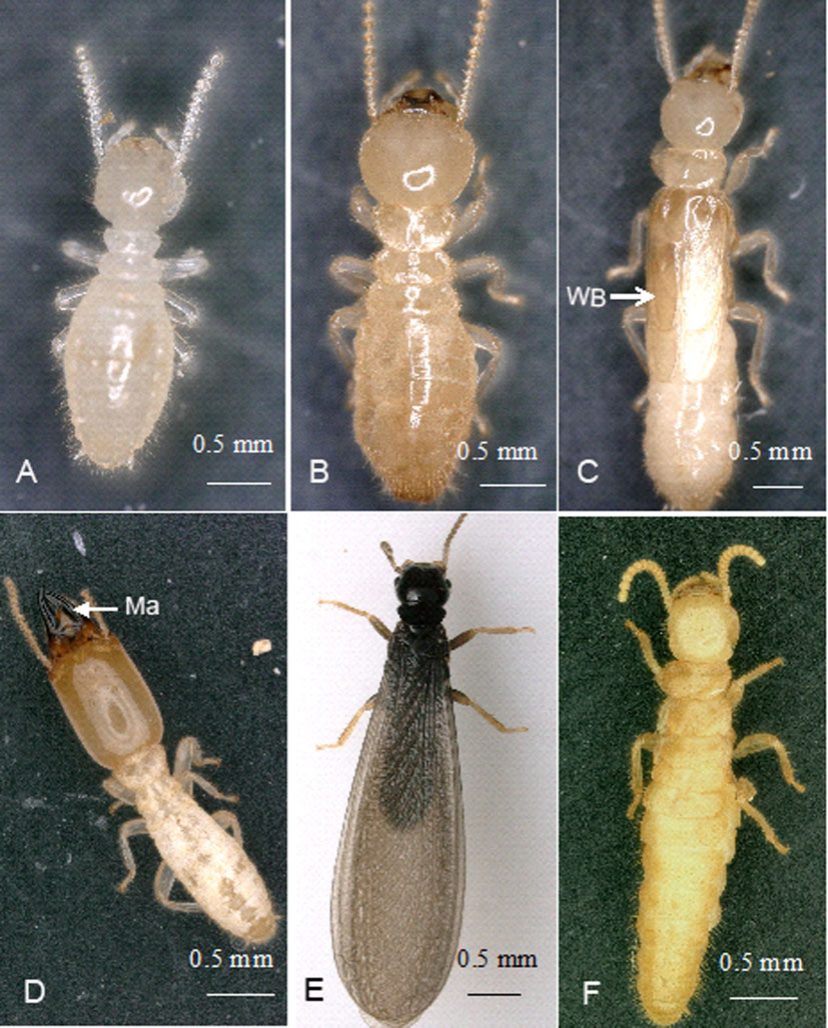
Morphological characteristics of individuals in the developmental pathway of *R. aculabialis*. (**A**) Larvae. (**B**) The workers had no wing buds compared with nymphs. (**C**) The nymphs had a white body and long wing buds (WB). (**D**) Soldiers with large heads and powerful mandibles (Ma). (**E**) The alates were characterized by darkened pigmentation, a hard cuticle, and black wings. (**F**) Apterous neotenic reproductive differentiation from workers had elongated abdomens and darker pigmentation. Scale bar = 0.5 mm.

The insect cuticle is an extracellular structure that acts as an exoskeleton and primary protective barrier against environmental stresses. The cuticle is made from 3 horizontal layers: the epicuticle, the exocuticle, and the endocuticle from outside to inside. The insect cuticle is a remarkable extracellular structure that is secreted by epidermal cells. The epicuticle and the exocuticle are secreted prior to ecdysis, and the endocuticle is generally formed after ecdysis (Koganemaru et al. 2013; Noh et al. 2017; Zhao et al. 2019). The cuticle provides protection against mechanical injury, pathogens, and parasites while also providing sensory perception, muscle attachment points, coloration, camouflage, and other physiological functions (Su et al. 2016; Noh et al. 2017). Since the cuticle is present at all developmental stages and essentially covers the entire body, it must exhibit great diversity in its physicochemical and mechanical properties to allow for growth and to accommodate the functions of the tissues and organs that are protected. Therefore, cuticles from different parts of the insect’s anatomy, or the same cuticle present at different developmental stages, may have different properties that arise, most likely from differences in chemical composition and molecular interactions, as well as the thickness and arrangement of morphologically distinct layers within the cuticle (Noh et al. 2017; Xiong et al. 2018).

Structural cuticular proteins (CPs) and chitin are the major components of the exocuticle and endocuticular layers. The most common insect cuticle-associated proteins belong to the CPR family. Previous studies have revealed that RR-1 of the CPR family is associated with soft (flexible) endocuticles, whereas RR-2 of the CPR family is more often associated with hard exocuticles. CPs are involved in virus–vector interactions. One of the RR-1 proteins is involved in cauliflower mosaic virus transmission (Deshoux et al. 2018). Apart from developmental processes, extrinsic factors may also regulate CP gene expression. Robust cuticular penetration resistance in the common bed bug correlates with increased steady-state transcript levels of CPR family genes (Koganemaru et al. 2013). CPs are sensitive to environmental stresses (Gallot et al. 2010; Xiong et al. 2018). In 3 cockroach species, *Gromphadorhina portentosa*, with the thickest cuticle, had the greatest tensile strength, strain energy storage, and puncture force resistance (Clark and Triblehorn 2014). Therefore, the expression of specific CPs is probably required for the formation of diverse cuticles at different developmental stages that exhibit appropriate combinations of physical, mechanical, and morphological properties to provide structural support, protection, and mobility.

Although a great deal of information is available regarding the structure of the termite cuticles, most studies focus on the exocuticle because it is easy to see the differences in sclerotized and pigmented exocuticles during caste development. Masuoka et al. (2013) have reported that gene expression changes in the tyrosine metabolic pathway regulate caste-specific cuticular pigmentation of termites, and high Laccase2 expression is likely involved in the formation of specific cuticular structures during soldier differentiation of the termite *R*eticulitermes *speratus.* They suggest that specific cuticular formation occurs in the exocuticles during caste differentiation (Masuoka et al. 2013; Masuoka and Maekawa 2016). However, until now, the developmental pattern of the endocuticle in termite individuals was unknown, and it is not clear whether the endocuticle is involved in pathways of caste differentiation.

In this study, to understand the relationship between endocuticle and caste differentiation and to reveal the cuticle as a model trait for caste-specific phenotypes, we compared the structural characteristics of the exocuticle and endocuticle of the larvae, nymphs, workers, soldiers, neotenic reproductives, and alates of termite *Reticulitermes aculabialis*. We sequenced the transcriptome of workers, soldiers, and alate adults (alates) and then identified the endocuticle protein genes. Finally, the expression patterns of the endocuticle protein genes determined by quantitative real-time PCR (qRT-PCR) were confirmed in 2 pathways (nymph line and worker line). These results provide new insight into the roles of endocuticles in caste differentiation and against environmental stresses.

## Materials and Methods

### Termites

The experimental termite *R. aculabialis* was collected from Northwest University, Xi’an, China. The last instar nymphs were collected in March (the last instar nymphs developed into alates via a moult in April), and late instar workers, soldiers, and alates were collected in May 2019 when the alates were swarming. Late instar workers were identified by the presence of 16 or more antennal segments. Apterous neotenic reproductives and larvae were collected in July 2019 when there were a lot of apterous neotenic reproductives and larvae in colonies. After collection from the field, samples were immediately fixed in Bouin’s solution for hematoxylin and eosin (HE) staining. Their head-thoraxes were cut and stored in liquid nitrogen until RNA extraction.

### Observation of exocuticle and endocuticle layers

The fixed samples were dehydrated in an ascending ethanol series and embedded in paraffin. Longitudinal sections, 7-μm thick, were collected on polylysine-coated slides. Deparaffifinized and rehydrated sections were stained in hematoxylin solution for 10 s and in eosin solution for 30 s. The sections were observed, and the thicknesses of the exocuticle and endocuticle in the heads, thoraxes, and abdomens were measured using a digital microscope (Keyence Company, Japan). Five replicates were used to obtain the average value of each individual (*N = *5). Significant differences were identified with the nonparametric Kruskal–Wallis test followed by Dunn’s multiple comparisons test. *P *<* *0.05 was considered significant.

### Transmission electron microscopy observation in workers

The heads and thorax–abdomens of workers were fixed in 0.1 M phosphate buffer (pH 7.0) containing 2% glutaraldehyde at 4°C for 4 h. After 3 30 min rinses in 0.1 M phosphate buffer (pH 7.0), samples were fixed overnight at 4°C in 1% osmium tetroxide and then rinsed 3 times for 30 min each in 0.1 M phosphate buffer (pH 7.0). Samples were dehydrated in a gradient ethanol series (30%, 50%, 70%, 85%, and 90% once each, then twice in 100%) at 4°C, with a final change in 1,2-epoxypropane, then embedded in Epon 812. Sections 0.08-μm thick were obtained using a Leica EMUC6 Ultramicrotome (Leica, Vienna, Austria). Sections were mounted on copper grids and stained with uranyl acetate and lead citrate. Observations were made using an H-7650 transmission electron microscopy (TEM) (Hitachi, Tokyo, Japan).

### cDNA library construction, illumina sequencing, and transcriptome assembly

The total RNA of the head thoraxes of workers, soldiers, and alates (*N = *20) was extracted using RNAiso Plus reagent (TaKaRa Bio. Inc., Japan). Oligo (dT) magnetic beads (Qiagen Co., Ltd., Shanghai) were used to extract polyA mRNAs for cDNA library construction. For first- and second-strand cDNA, random hexamer primers, and buffers, dNTPs, RNase H, and DNA polymerase I were used, followed by cDNA synthesis, and then the mRNAs were shattered into small fragments. The cDNA fragments were attached to the sequencing adaptors after their refinement. The cDNA was checked by using agarose gel electrophoresis, with a fragment length of 200 bp. After refinement, PCR was used to build a final cDNA library. Illumina sequencing platform technology (Illumina HiSeq TM2500) (Guangzhou, China) was used to sequence the previously built cDNA. Practical extraction and report language (Perl) software was used to filter the clean reads and adaptor sequences read. Transcriptome *de novo* assembly was constructed by using Trinity software (version 2.0.6). The expression level of a single gene was computed by the total number of reads that were covered by that gene and the expression quantity of all exposed genes was computed by the same method as above. Unigenes were annotated by using Nr (non-redundant protein) annotation results. Cellular components, molecular function, and biological processes were the 3 main groups of gene ontology, which contained 51 functional categories. In differentially expressed genes, the KEGG and Gene Ontology pathways with *Q* values < 0.05 were remarkably improved. Blast2GO software was used to analyze the annotated unigenes of gene ontology. WEGO software was used for functional classification of unigenes.

### Gene expression analysis

RNA was extracted from the head–thoraxes of workers, soldiers, nymphs, neotenic reproductives, and alates using RNAiso Plus reagent (TaKaRa Bio. Inc., Japan) (*N = *20). qRT-PCR was used to amplify complementary DNA by using 50 ng of mRNA and prime script RTase (Takara Bio. Inc., Japan). SYBR Premix Ex TaqTM II (Takara. Bio. Inc., Japan) was used in the quantitative reaction with Light Cycler 480 software 1.2.0.0625 (Roche Diagnostic, Switzerland). The beta-actin gene was used as a reference for expression results and Ct values were used to maintain all reactions in qRT-PCR. The 2^−ΔΔCt^ method was used to measure the corresponding gene expression. All qRT-PCR experiments were repeated in 3 biological and 3 technical replications. We used the nonparametric Kruskal–Wallis test combined with *post hoc* Dunn’s multiple test to compare the significant differences among workers, soldiers, nymphs, neotenic reproductives, and alates.

## Results

### Caste differentiation with changes in morphology

In the process of caste development of *R. aculabialis* (Figure 1), only the cuticle of alate adults (alates) changed dramatically in appearance. The alates that developed from white last instar nymphs were characterized by darkened pigmentation, a hard cuticle, and black wings. Workers, soldiers, and neotenic reproductives retained a light cuticle and soft body. However, the soldiers that differentiated from workers had large, highly sclerotized heads, and powerful mandibles (Figure 2).

### The change in thickness of exocuticle layers in caste differentiation

**Figure 3.**
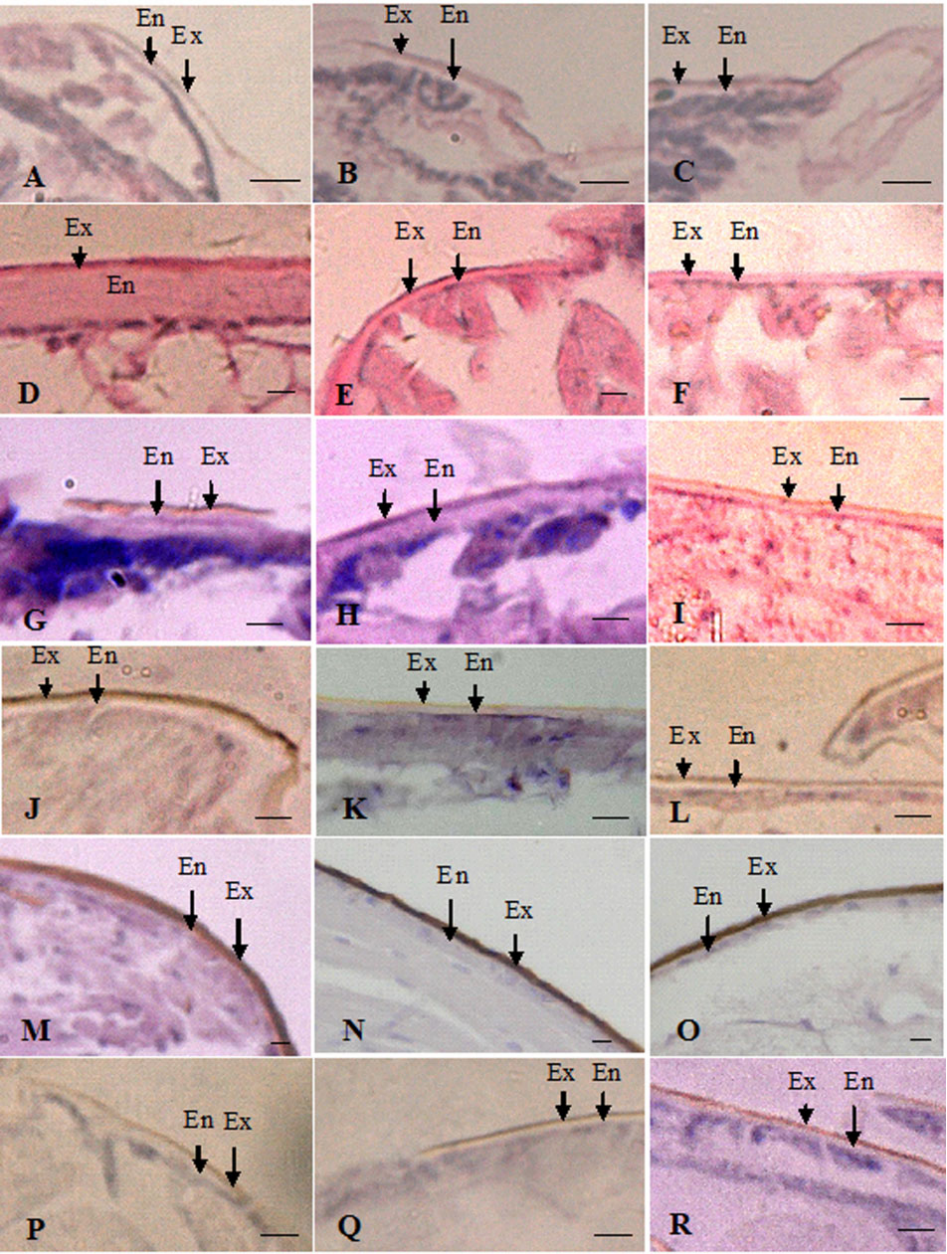
The morphology of exocuticle and endocuticle layers in the heads, thoraxes, and abdomens of larvae, nymphs, alates, workers, soldiers, and neotenic reproductives of *R. aculabialis*. (**A–C**) The head, thorax, and abdomen of the larva, respectively. (**D**) Thickest endocuticle layer in the worker head. (**E** and **F**) Thin endocuticle layer in the worker thorax and abdomen compared with the head. (**G**–**I**) The head, thorax, and abdomen of the nymph, respectively. (**J**–**L**) The head, thorax, and abdomen of the soldier, respectively. (**M**–**O**) Thickest exocuticle and thinnest endocuticle in the head, thorax, and abdomen of the alate, respectively. (**P**–**R**) The head, thorax, and abdomen of the neotenic reproductive, respectively. Ex, exocuticle; En, endocuticle. Scale bar = 10 μm.

**Figure 4.**
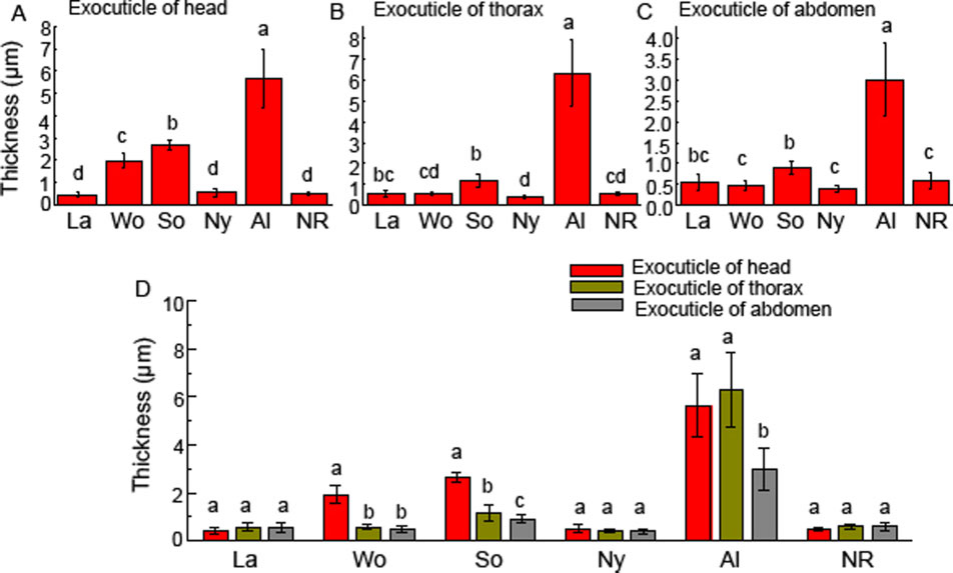
The thickness of exocuticle layers in the heads, thoraxes, and abdomens of larvae, nymphs, alates, workers, soldiers, and neotenic reproductives of *R. aculabialis*. (**A**) The thickness of exocuticle layers in the heads of larvae, nymphs, alates, workers, soldiers, and neotenic reproductives. (**B**) The thickness of exocuticle layers in the thoraxes. (**C**) The thickness of exocuticle layers in the abdomens. (**D**) A complete comparison on the thickness of the exocuticle layer among the head, thorax, and abdomen. Different alphabets on each bar indicate the significant difference (*P* < 0.05). La, larvae; Wo, workers; So, soldiers; Ny, nymphs; Al, alates; and NR, neotenic reproductives.

The thickness of the exocuticle layers of larvae, nymphs, neotenic reproductives, alates, workers, and soldiers was measured. The exocuticle was pigmented and the endocuticle was not pigmented. Therefore, the different cuticle colors in individuals were related to the thickness of the exocuticle (Figure 3). The thickest exocuticle layers occurred in the heads and thoraxes of alates, which were 5.68 ± 1.30 and 6.31 ± 1.56 µm thick, respectively, and showed no significant difference in thickness between them (*P > *0.05). The exocuticle layers in the head–thoraxes of alates were approximately 2-fold thicker than those in the abdomens (3.01 ± 0.89 µm). The exocuticle of alates comprised the largest percentage of cuticle (80%). Exocuticles were thicker in hard-bodied alates and thinner in soft-bodied nymphs, workers, soldiers, and neotenic reproductives (Figure 4).

The thinnest exocuticle layers were found in larvae, nymphs, neotenic reproductive, and thorax–abdomens of workers, and there was no significant difference in thickness among them. In nymphs and neotenic reproductives, there was no significant difference in thickness among their head, thorax, and abdomen (Figure 4).

Obviously, there was a great difference in the exocuticle structural characteristics among different tagmata of workers and soldiers. The heads of workers (1.96 ± 0.34 µm) and soldiers (2.68 ± 0.21 µm) had thicker exocuticle layers, and there was a significant difference in the thickness of the exocuticle between the heads of workers and soldiers (*P > *0.05). The exocuticle layer in the heads of soldiers was approximately 3-fold thicker than that in their thoraxes (1.18 ± 0.33 µm) and abdomens (0.91 ± 0.16 µm). In 3 tagmata of workers, the thickest exocuticle layer was in the heads. The exocuticle layer in the heads of workers was approximately 4-fold thicker than that in their thoraxes (0.56 ± 0.07 µm) and abdomens (0.48 ± 0.11 µm), respectively. There was no significant difference in the thickness of the exocuticle among the nymphs, neotenic reproductives, and thorax–abdomens of workers (Figure 4).

### The change in the thickness of endocuticle layers in caste differentiation

**Figure 5.**
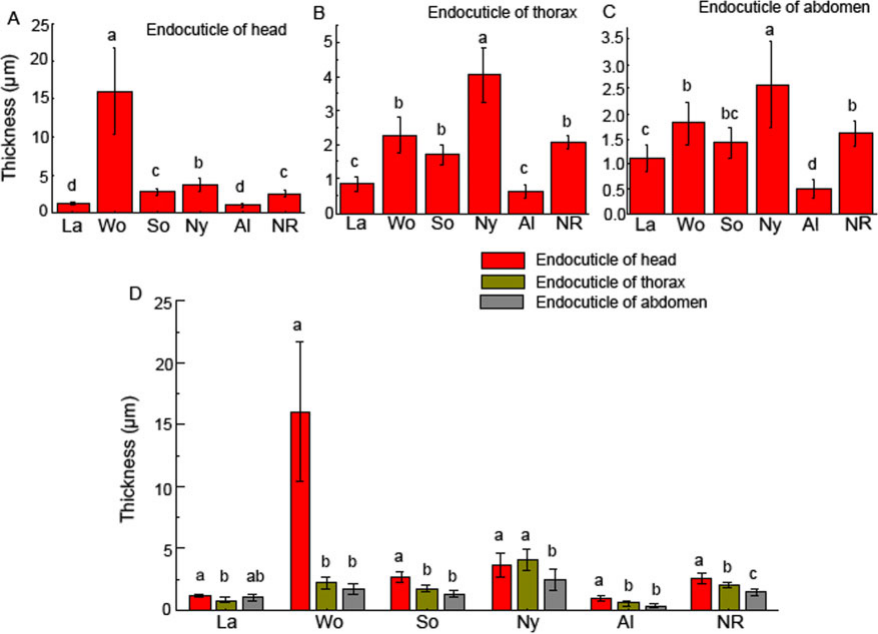
The thickness of endocuticle layers in the heads, thoraxes, and abdomens of larvae, nymphs, alates, worker soldiers, and neotenic reproductives of *R. aculabialis*. (**A**) The thickness of endocuticle layers in the heads. (**B**) The thickness of endocuticle layers in the thoraxes. (**C**) The thickness of endocuticle layers in the abdomens. (**D**) A complete comparison on the thickness of the endocuticle layer among the head, thorax, and abdomen. Different alphabets on each bar indicate the significant difference (*P *<* *0.05). La, larvae; Wo, workers; So, soldiers; Ny, nymphs; Al, alates; and NR, neotenic reproductives.

**Figure 6.**
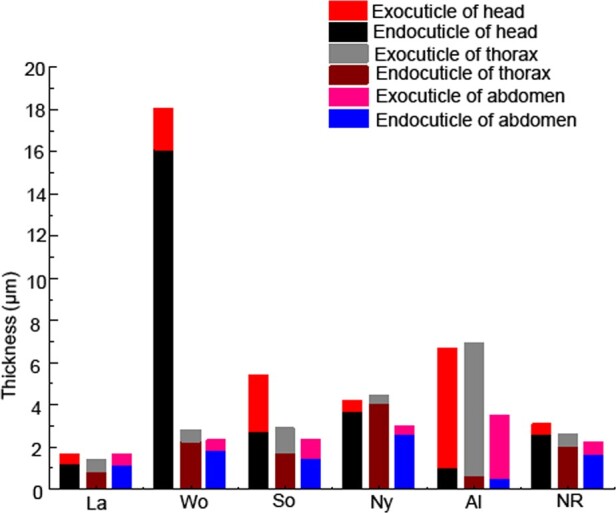
The combination of exocuticle and endocuticle layers showed the change pattern of cuticle in caste differentiation of *R. aculabialis.* The thickest cuticle in the heads of the workers.

Our data showed that the thickest endocuticle layers were found in the heads of the workers (16.07 ± 5.68 µm), and in workers, there was a great difference in the endocuticle thickness between heads and thorax–abdomens. The thickness of the endocuticle in the heads of workers was approximately 7-fold thicker than that in the thoraxes (2.26 ± 0.52 µm) and abdomens (1.82 ± 0.42 µm). The endocuticle in the heads of workers comprised the largest percentage of cuticle (84%). However, there were no significant differences in the thickness of the endocuticle between heads and thorax–abdomens in larvae and nymphs (Figure 5). The thickest cuticle in the workers was largely due to the endocuticle thickness of their heads (Figure 6).

The thinnest endocuticle layers occurred in the head, thorax, and abdomen of alates, which were 0.96 ± 0.18, 0.62 ± 0.22, and 0.48 ± 0.18 µm thick, respectively. Although alates were differentiated from last instar nymphs via a moult, the thickness of the endocuticle in the head, thorax, and abdomen of nymphs was approximately 4-fold, 6-fold, and 5-fold thicker than that in alates, respectively.

After the larvae developed into workers and nymphs, the thickness of the endocuticle increased significantly, and there was a significant difference in the thickness of the endocuticle between workers and nymphs. The endocuticle layer in the heads of the workers was approximately 4-fold thicker than that of the nymphs (3.65 ± 0.98 µm), whereas the endocuticle in thorax–abdomens of nymphs was significantly thicker than that of workers. Interestingly, after the nymphs developed into alates, the thickness of the endocuticle decreased by 70%.

The soldiers and neotenic reproductives were differentiated from workers via 2 moults (Su et al. 2016). Compared with the heads of workers, the thickness of the endocuticle in the heads of soldiers and neotenic reproductives was thinner. The thickness of the endocuticle in the head of the workers was approximately 6-fold and 4-fold thicker than that of soldiers and neotenic reproductives, respectively. However, there were no significant differences in the thickness of the endocuticle of the thorax–abdomens among neotenic reproductives, workers, and soldiers (Figure 5).

### Ultrastructure of endocuticle layers in workers

**Figure 7.**
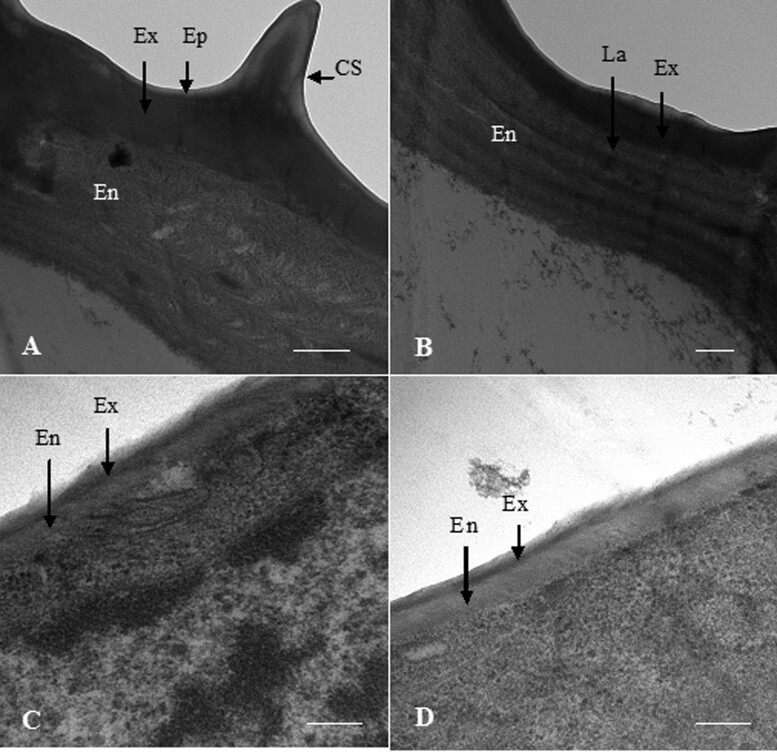
Ultrastructure of endocuticle layers in workers by TEM. (**A**) The surface of the head of worker had conical spikes. Scale bar = 5 μm. (**B**) The endocuticle of the head consisted of 5 thick brick-like horizontal laminae. Scale bar = 5 μm. (**C**) The endocuticle of the thorax. Scale bar = 5 μm. (**D**) The endocuticle of the abdomen. Scale bar = 4 μm. Ep, epicuticle; Ex, exocuticle; En, endocuticle; CS, conical spikes; and La, laminae.

Based on the ultrastructure from TEM observation of workers, the epicuticle, endocuticle, and exocuticle were clearly differentiated. The epicuticle was the outermost and thinnest lay. We found that the surface of the heads of workers had conical spikes and the endocuticle of the heads consisted of 5 thick brick-like horizontal laminae. Each lamina exhibited a similar degree of electron density. However, the thorax–abdomen had no conical spikes, and a well-ordered lamellar structure did not exist in the endocuticle of thorax–abdomens. The endocuticle in the heads was approximately 7-fold thicker than that in the thoraxes and abdomens. However, in both heads and thorax–abdomens, the endocuticle accounted for approximately 4/5 of the total thickness of the cuticle (Figure 7).

### Endocuticle protein genes of *R. aculabialis*

Based on the protein annotation results, our analysis of the RNA-seq data of *R. aculabialis* identified 6 endocuticle protein genes that were similar to the endocuticle protein genes of termite *Zootermopsis nevadensis* (Table 1) and 4 predicted endocuticle protein genes that were similar to the endocuticle protein genes of *Cimex lectularius* and *Bemisia tabaci*. These endocuticle proteins were endocuticle structural glycoproteins SgAbd-2, SgAbd-9, Abd-5, SgAbd-2-like, Abd-4-like, and SgAbd-1-like, which were classified into the RR-1 group of the CPR family (CPs with the R&R consensus). Previous studies have suggested that RR-1 proteins are associated with soft (flexible) endocuticles. Therefore, in *R. aculabialis*, these RR-1 proteins were related to the formation of endocuticles. All raw sequence reads have been deposited in the NCBI SRA database and are accessible through SRA accession number SRP199695. The assembled gene sequences have been deposited in the NCBI TSA database under accession number GHMS00000000.

**Table 1.** Six endocuticle protein genes of *R. aculabialis* were identified and annotated

Unigene ID	Annotation
Unigene 0001094	Endocuticle structural glycoprotein SgAbd-9 [*Z. nevadensis*]
Unigene 0001350	Endocuticle structural glycoprotein SgAbd-2 [*Z. nevadensis*]
Unigene 0006451	Endocuticle structural glycoprotein SgAbd-2 [*Z. nevadensis*]
Unigene 0034952	Endocuticle structural glycoprotein SgAbd-2 [*Z. nevadensis*]
Unigene 0003968	Endocuticle structural glycoprotein ABD-5 [*Z. nevadensis*]
Unigene 0016014	Endocuticle structural glycoprotein SgAbd-9 [*Z. nevadensis*]

### Endocuticle protein gene expression in nymphs, workers, soldiers, alates, and neotenic reproductives

**Figure 8.**
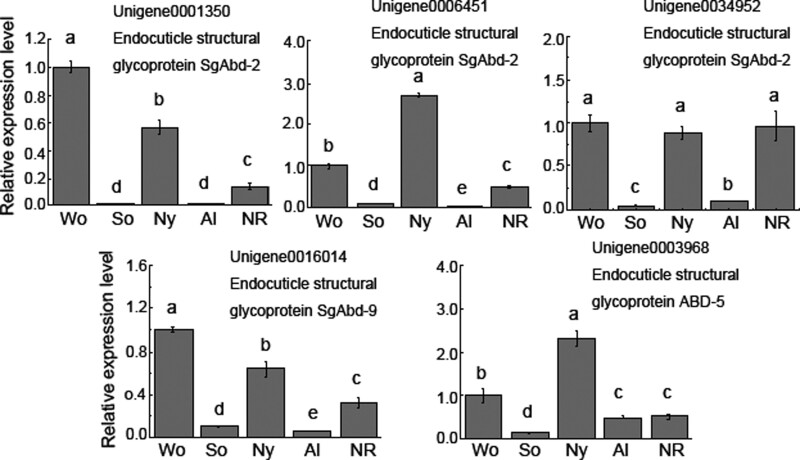
The relative expression levels of the endocuticle protein genes in nymphs, alates, workers, soldiers, and neotenic reproductives of *R. aculabialis* by qRT-PCR analysis. Wo, workers; So, soldiers; Ny, nymphs; Al, alates; and NR, neotenic reproductives. Different alphabets on each bar indicate the significant difference (*P* < 0.05).

We compared the expression profiles of 5 endocuticle protein genes (3 *SgAbd-2*, 1 *SgAbd-9*, and 1 *ABD-5*) in the workers, nymphs, soldiers, alates, and neotenic reproductives by qRT-PCR (Figure 8). No expression of 1 *SgAbd-9* (unigene0001094) was detected in our qRT-PCR analyses. In worker, soldier, and reproductive (alate and neotenic reproductive) castes, all 5 endocuticle protein genes were expressed at higher levels in the workers than in the alates and soldiers. The expression levels of 3 *SgAbd-2* genes, *SgAbd-9*, and *ABD-5* in workers were approximately 71-, 8-, 23-, 9-, and 7-fold higher than those in soldiers, respectively, and were approximately 55-, 66-, 11-, 16-, and 2-fold higher than those in alates, respectively. Four endocuticle protein genes were expressed at higher levels in the workers than in the neotenic reproductives that developed from workers, and there was no significant difference in the expression of 1 *SgAbd-2* gene (unigene 0034952) between workers and neotenic reproductives. The expression levels of 4 endocuticle protein genes in neotenic reproductives were significantly higher than those in alates; there was no significant difference in the expression level of ABD-5 (Unigene0003968) between them. The expression levels of 3 *SgAbd-2* and *SgAbd-9* in neotenic reproductives were approximately 7-, 33-, 11-, and 22-fold higher than those in alates, respectively.

The expression levels of endocuticle protein genes decreased after the nymphs developed into alates via a moult. All 5 endocuticle protein genes were expressed at higher levels in the nymphs than in the alates. The expression levels of 3 *SgAbd-2*, *SgAbd-9*, and *ABD-5* in nymphs were approximately 31-, 181-, 10-, 9-, and 4-fold higher than those in alates, respectively. Similarly, the expression levels of endocuticle protein genes greatly decreased after workers developed into neotenic reproductives or soldiers via 2 moults.

## Discussion

Our histological data demonstrate that the endocuticle and exocuticle are dynamic structures in caste development of *R. aculabialis*, and the first difference in cuticle structure occurred after larvae differentiation bifurcates into workers and nymphs. The endocuticle layer and exocuticle layer in the heads of the workers were approximately 4-fold thicker than those of the nymphs, respectively. In *Reticulitermes*, nymphs follow the reproductive pathway to develop into alates, which are the primary reproductives that swarm to form new colonies, and the worker pathway, which generally leads to worker termites (Su et al. 2017). Workers forage for food (wood), build and repair colonies, and tend other individuals in colonies. The workers do all the work with their heads. There is no doubt that all of the eggs, larvae, nymphs, reproductives, and soldiers would die without workers’ help in a termite colony (Korb and Hartfelder 2008; Bignell et al. 2011). Our findings are strong evidence that the cuticle structure of heads of termites is related to the differentiation of reproductive and non-reproductive pathways.

We found that workers exhibited the thickest endocuticle, resulting in the thickest cuticle in the workers and ameliorating cuticle performance. In 3 cockroach species, *G. portentosa* with the thickest cuticle had the greatest tensile strength, strain energy storage, and puncture force resistance, while *Periplaneta americana* with the thinnest cuticle had the weakest values (Clark and Triblehorn 2014). Moreover, thickening of the bed bug cuticle may contribute to decreased insecticide penetration (Koganemaru et al. 2013). We believe that the thicker endocuticle of termite workers is specialized and has special functions. The characteristics of the cuticle are mainly determined by exocuticle and endocuticle deposition. Generally, a major portion of CPs in insects is enclosed in the exocuticle and endocuticle, which provides muscular support and acts as a protective shield. Several layers of proteins and fibrous chitins that cross each other in a sandwich motif are combined to form a soft and flexible endocuticle (Dittmer et al. 2015; Vannini and Willis 2017). In *Reticulitermes*, newly hatched individuals (larvae) develop into workers or nymphs via moults. The workers have unique flexibility in that a worker can develop into older instar workers, sterile soldiers, or neotenic reproductives. Surprisingly, we found that after a worker developed into a soldier or neotenic reproductive, the thickness of the endocuticle decreased significantly. We suggest that the structure of endocuticles can be changed in the process of caste differentiation.

Obviously, the thinning of the endocuticle and thickening of the exocuticle were 2 significant events from nymphs developing to alates. Unlike exocuticle thickening, it is surprising that the alates have the thinnest endocuticle. Alates interact with the outside environment much more than the other castes. The alates have the thinnest endocuticle and the thickest exocuticle, which increase the hardness of the body wall, facilitating joint movement of the muscle and pulling up the wings to fly outside of their colony. Muscles are connected to the body wall along with attachment fibers regulating throughout the epicuticle and cuticle. The movement of different parts of the body and appendages, such as the wings, depends on muscle attachment (Moussian 2010). Therefore, we infer that the alates have the thinnest endocuticle layer to reduce weight and strengthen muscle attachment for flight.

The thickness of the endocuticle in the heads of workers was approximately 7-fold thicker than that in the thorax–abdomens. The head of an insect is an important part of the body with a highly sclerotized and thick exoskeletal head capsule to protect their brain. The head capsule of insects contains the bulk of vital sensory organs. The sensory organs on the cuticle are sensitive to touch, sound, and vibration and control the defense system (Romoser and Stoffolano 1999). In a previous study of *Locusta migratoria*, the whole-body transcriptome showed that RR-1 protein gene expression was very high in the head, and the expression of these genes is continuously involved in cuticle synthesis (Zhao et al. 2017). We consider that the thickest endocuticle occurs in the head of workers because the head is the center of the sensory nerves, and the endocuticle is also related to sensory factors. We found that the surface of the heads of workers had conical spikes, and the endocuticle of the heads consisted of 5 thick brick-like horizontal laminae. However, the thorax–abdomen had no conical spikes, and lamellar structure did not exist in the endocuticle of thorax–abdomens. The head of workers is hardened, but the body is not. The workers use their mouthpart to dig tunnels and are responsible for defending the colony from invaders. The heads with conical spikes can dig tunnels more efficiently, thick endocuticle layers and 5 thick brick-like horizontal laminae can bear pressure and protect their heads. Therefore, the worker can benefit from the added protection by a thick endocuticle in the head.

We identified 3 endocuticle structural glycoproteins (SgAbd-2, SgAbd-9, and Abd-5) in *R. aculabialis* and classified them into the RR-1 group of the CPR family (CPs with the Rebers and Riddiford consensus) (Andersen 1998; Willis 2010). Therefore, SgAbd-2, SgAbd-9, and Abd-5 are involved in endocuticle synthesis of *R. aculabialis*. CPR is the largest CP family in arthropods and includes 2 major groups (RR-1 and RR-2) and a very minor RR-3 group. RR-1 proteins are associated with soft (flexible) endocuticles, whereas RR-2 proteins are associated with hard exocuticles. The majority of CPR genes have transcripts that are present in both the pharate and post-eclosion stages (Willis 2010; Vannini and Willis 2017). In addition, it was reported by phylogenetic analysis that in the locust *L. migratoria*, LmAbd9 has a close relationship with SgAbd-9. After injection of ds*LmAbd-9* into fifth instar nymphs, although the nymphs could moult to adults, the endocuticle of adults was thinner, and there were significantly fewer endocuticular lamellae than the control (Zhao et al. 2019).

All individuals in the colony have the same genetic information, and thus, the regulation of gene expression is crucial for the formation of caste-specific phenotypes (Masuoka and Maekawa, 2016). In this study, we found that the endocuticle protein genes in different castes showed specific expression patterns. The endocuticle protein genes were expressed at higher levels in the workers than in the reproductives and soldiers, which was consistent with the thickest endocuticle in workers. Much of the cuticular diversity is due to the variation in protein composition entailing differences in the molecular architecture of the procuticle (exocuticle and endocuticle) (Andersen 2010; Pan et al. 2018). Thus, the combination of different kinds of endocuticle proteins of termites results in the construction of caste-specific endocuticles. Differential expression of various CP genes can produce cuticle layers with different physical properties at different stages, indicating that CP genes have been adapted to the characteristic lifestyle of various insects (Liu et al. 2019). Previous qRT-PCR analysis indicated that the transcripts of RR-1 were upregulated in resistant bed bugs (Koganemaru et al. 2013). CP mutation of the silkworm *Bombyx mori* can lead to deficiency in resources required to construct the cuticle, resulting in a thin cuticle and reduced resistance to UV and insecticides (Xiong et al. 2018).

Our results revealed that the endocuticle protein genes displayed different expression patterns between the primary reproductives (alates) and secondary reproductives (neotenic reproductives), in which the expression levels of endocuticle protein genes in the alates were significantly lower than those in neotenic reproductives. In the locust *L. migratoria*, the expression level of endocuticle protein *LmAbd9* did not decrease after nymphs moulted to adults (Zhao et al. 2019). However, in *R. aculabialis*, the expression levels of all endocuticle protein genes in alates were extremely low after the nymphs developed into alates. Therefore, we infer that low expression of endocuticle protein genes in alates leads to thin endocuticle lays, which may be related to the unique function of alates, especially flight.

The workers of *Reticulitermes* have unique flexibility in that a worker has the capability to develop into other castes (neotenic reproductives and soldiers). We found that the expression level of endocuticle protein genes decreased significantly after workers developed neotenic reproductives and soldiers. Previous studies have reported that viruses bind to specific retention sites on the cuticle and that virus-interacting molecules are cuticle proteins (Deshoux et al. 2018). Termite nests and their surrounding soils are laden with a variety of potential pathogens, including bacteria, nematodes, viruses, protozoa, and rickettsiae. Workers may control microclimatic conditions within their nests, making it ideal not only for the development of offspring but also for the growth of microorganisms (Bignell et al. 2011). Obviously, the endocuticle protein genes are expressed in a caste-specific manner, indicating that immune mechanisms of endocuticle in workers may have to adapt to particular sets of pathogens. We infer that the endocuticle is an important factor in workers becoming nest builders and provides an effective barrier from the natural environment.

## Accession Numbers

All raw sequence reads have been deposited in the NCBI SRA database and are accessible through SRA accession number SRP199695. The assembled gene sequences have been deposited in the NCBI TSA database under accession number GHMS00000000.

## Authors’ contributions

X.S. and C.Y. designed the study and wrote the manuscript. C.Y., Z.S., T.W., and N.u.S. performed the experiment. C.Y. analyzed the results. W.Z. and L.X. collected the termites. All authors read and approved the final manuscript.
